# Evaluating the validity and reliability of the Chinese entrapment scale and the relationship to depression among men who have sex with men in Shanghai, China

**DOI:** 10.1186/s12888-021-03333-9

**Published:** 2021-07-02

**Authors:** Chen Xu, Xiaoyue Yu, Lhakpa Tsamlag, Shuxian Zhang, Ruijie Chang, Huwen Wang, Shangbin Liu, Ying Wang, Yong Cai

**Affiliations:** School of Public Health, Shanghai Jiao Tong University School of Medicine, No.227, South Chongqing Road, Shanghai, 200025 People’s Republic of China

**Keywords:** Men who have sex with men, Entrapment scale, Reliability, Validity, Depression, China

## Abstract

**Background:**

Perception of entrapment can emerge when someone feels trapped in an aversive situation and incapable of escape. Depression is closely related to the construct of entrapment. In China, men who have sex with men (MSM) have a high prevalence of depression; therefore, a tool to evaluate entrapment in this population is needed. We evaluated the validity and reliability of the Chinese version of the entrapment scale (ES) and the relationship to depression among MSM in Shanghai, China.

**Methods:**

We recruited 304 MSM from four districts in Shanghai, China. Participants completed health behavior questionnaires that included baseline information and psychological measurements such as the ES and Patient Health Questionnaire (PHQ-9). The sample was randomly divided into two groups for exploratory factor analysis (*n* = 143) and confirmatory factor analysis (*n* = 161). Criterion validity was tested to explore the correlation between the ES and PHQ-9 scores. The reliability of the ES was evaluated with internal consistency reliability (Cronbach’s α coefficient) and split-half reliability (Spearman-Brown coefficient). We performed hierarchical regression analysis to determine the variance explained of entrapment to predicting depressive symptoms after adjusting for sociodemographic factors. Finally, receiver operator characteristic curve analysis was performed to measure the optimal ES cut-off value for predicting depression.

**Results:**

Factor analysis showed the ES had one principal component, and one-dimensional scale had more acceptable model fit indices than two-dimensional model. The correlation coefficient between the ES and PHQ-9 scores was 0.756 (*P* < 0.01). The Cronbach’s α coefficient was 0.970 and the Spearman-Brown coefficient was 0.976. ES scores significantly predicted an additional 45.1% of depressive symptoms after controlling for sociodemographic characteristics in the MSM population (β = 0.689, *P* < 0.001). The optimum cut-off value was 23, which had a sensitivity of 70% and a specificity of 85.4%.

**Conclusions:**

The Chinese version of the ES has good validity and reliability in the MSM population in Shanghai, and can be used to evaluate perception of entrapment among MSM. The findings confirmed an association between entrapment and depression.

**Supplementary Information:**

The online version contains supplementary material available at 10.1186/s12888-021-03333-9.

## Background

The concept of entrapment originates from ethological studies concerning blocked or arrested defensive behaviors of animals to escape from social threats and stressors (e.g., fight, flight, or both) [1]. When encountering problems that cannot be accepted or are relatively novel, fight or flight strategies may not achieve expected results, and psychological disorders may emerge along with defeat [2]. Defeat represents a sense of failed social struggle, losing social status, powerlessness or missing personal goals [3]. Defeat, as well as entrapment, plays a central role in the development of psychopathology in the human being [4]. Feeling defeated and trapped (called arrested flight) may lead individuals to perceive entrapment, which is considered more serious than being defeated but able to escape [2].

Gilbert and Allan defined entrapment as a personal feeling in which an individual is in an adverse state or environment and has a strong motive to take flight or get rid of the stressor, but is incapable of escape. In the social rank theory, those who have lost their status are at greater risk of pathology. Based on the social rank theory, Gilbert and Allan emphasized that entrapment played an important role in the social rank theory of depression and increased significantly to the explained variance of depression after adjusting for other social rank factors [5]. Entrapment can occur following long-term, stressful life events or situations and may be associated with the onset of depressive disorders. Previous studies have shown clear and robust correlations between entrapment and depression and attributed the occurrence of depression to the perception of entrapment [5–9]. Furthermore, feeling of entrapment and desire to escape have also been strongly linked to suicide ideation [10–13]. The Cry of Pain Model suggested that in a sample of first-time and repeat self-harm patients, entrapment had a mediating role in the defeat-suicide ideation relationship [14]. A previous systematic review reported that self-perceived defeat and entrapment played key roles in depression, anxiety, suicide ideation, and post-traumatic stress disorder, and emphasized that entrapment played a decisive role in depression [8].

Gilbert and Allan developed the entrapment scale (ES) in 1998 to measure subjective experiences of entrapment. The scale was divided into two domains based on the causes of entrapment: external entrapment (EE) and internal entrapment (IE). EE relates to perception of things in the outside world that induce escape motivation; for example, being trapped in a relationship or a lack of resources. IE relates to escape motivation triggered by internal feelings and thoughts [5, 15]. Gilbert and Allan conducted a study and recruited two groups of undergraduate students and patients with depression and the study showed that both EE and IE had satisfactory internal consistency and could be considered unidimensional measures [3]. That study also showed the variables of entrapment performed well and presented robust correlations with depression and hopelessness in both groups [5]. The original ES has been translated into multiple languages since its publication and has been shown to have good reliability and validity in different populations [7, 16, 17]. EE and IE were originally conceptualized as two distinct constructs and evaluated using two subscales. However, Taylor et al. suggested entrapment may be better considered as a single factor [18]. This was also verified by reliability and validity evaluations of the German versions of the ES [7]. Whether the ES has a single-factor structure or a two-factor structure remains to be explored.

The ES has been found to be applicable in different populations, including healthy subjects, patients with depression, caregivers, and medical students. However, no study has evaluated perception of entrapment among men who have sex with men (MSM), which is a male population performing sexual behavior with other males regardless of their self-identified sexual orientations (for example gay/homosexual, heterosexual or bisexual) [19]. MSM is a sexual minority with a high prevalence of mental health problems including depression [20–22]. Most studies with MSM have focused on high-risk sexual behavior, HIV infection, and substance use rather than mental health. However, in China, MSM has been marginalized because of their sexual orientation and corresponding prejudice related to traditional briefs, and are generally not understood or accepted by the public. MSM are subject to social stress, prejudice, exclusion, and physical and verbal violence, which seriously affects their daily life and physical health; they also suffer more psychological pressure, anxiety, depression, and panic disorders than heterosexual men [23–25]. In addition, the presence of current psychiatric disorders has a significant independent effect on suicide ideation among MSM [26]. In Shanghai, China, nearly one-third (30.9%) of MSM suffer from depression, which is far higher than the prevalence of depression among adults in general (2.06%) [27]. A previous study reported 10.6% of the MSM sample had suicide ideation in the past year [28]. Given the relationship between entrapment, depression, and suicide ideation, an instrument to measure perception of entrapment among MSM in China is worth exploring. With the permission of the authors of the original scale, our team translated the scale into Chinese and firstly verified its reliability and validity in medical student [29]. However, the Chinese version of the ES has not been applied in the MSM population.

The present study aimed to: 1) test the reliability and validity of the Chinese version of the ES; 2) explore the proportion of variance in explaining depressive symptoms; and 3) calculate the optimal cut-off value of the ES for predicting depression among MSM in Shanghai, China.

## Methods

### Study population and eligibility criteria

A cross-sectional study was conducted from March to November 2018 in four districts of Shanghai. After removing missing data, a total of 304 participants were included in the analyses. The inclusion criteria were: biological males aged over 18 years who had engaged in sexual behavior with men over the past 6 months. The exclusion criterion was participants with mental or cognitive impairment, unconsciousness so that they cannot verbalize their real feelings or fill out questionnaires.

### Recruitment and study procedure

The hidden nature of MSM prevented this study from conducting a large-scale investigation with random sampling; therefore, a snowball sampling method was used [30, 31]. First, 5–10 eligible MSM were selected as initial “seeds” in each district with the help of the local Center for Disease Control and Prevention and non-governmental organizations. Then, these participants were tasked with recruiting eligible subjects from the same sociocultural background. These second groups of participants were also asked to provide information on other potential subgroup participants, and this process continued until no sample could be found through snowball sampling method.

The investigators were gathered together and trained to understand the survey content, methods, and relevant precautions. The investigators reached consensus on the health behavior questionnaire. Anonymous face-to-face interviews with participants were conducted as follows. First, the investigators explained the goal and procedure of the survey to participants in detail, answered any questions, and obtained their written informed consent. Next, each participant was asked to independently complete a self-administered questionnaire in a private room. The questionnaire took around 30 min to complete. After completion of the questionnaires, the investigators performed integrity checks and logic checks for each questionnaire, and resolved any problems in a timely manner to ensure accuracy of the collected data.

### Entrapment scale (ES)

The 16 items of the Chinese version of the ES are divided into external entrapment (items 1–10) and internal entrapment (items 11–16). The response options for each item are “not at all,” “a little bit,” “moderately,” “quite a bit,” and “extremely,” which correspond to scores of 0–4. The total score ranges from 0 to 64. A higher score indicates a stronger sense of entrapment. The final Chinese version of the ES is detailed in Additional files 1.

### Patient health Questionnaire-9 (PHQ-9)

The PHQ-9 was developed for criteria-based screening and diagnosis of depression [32]. The scale has been widely applied in primary care settings and demonstrated acceptable psychometric properties [33–35]. Compared with other commonly used clinical depression assessment tools, the PHQ-9 has the advantages of having fewer items, being easier to understand, and less time-consuming. The scale comprises nine items that evaluate the frequency of depressive symptoms in the previous 2 weeks. Each item is scored from 0 to 3 (representing “not at all”, “a few days”, “more than half a day” and “nearly every day”). Total scores range from 0 to 27. The optimal cutoff point is ≥10, which was described as diagnostic depression in a systematic review [36]. Many studies have confirmed that the generation of entrapment and defeat may trigger depression and lead to poor psychological states such as lack of self-esteem and self-confidence [5, 8, 9, 37]. Therefore, this study used the PHQ-9 to assess depressive symptoms among MSM, consistent with previous studies [38, 39]. The internal consistency reliability (Cronbach’s α coefficient) of the PHQ-9 in this study was 0.874.

### Statistical analyses

Participants’ sociodemographic characteristics were calculated by numbers and proportions. The ES scores were described as mean ± standard deviation (SD) and median (inter-quartile range, IQR). A histogram of the ES scores was provided in Additional file 2. Differences between sample subgroups were tested with non-parametric tests. Participants were randomly divided into two groups using a random number generator to perform exploratory factor analysis (*n* = 143) and confirmatory factor analysis (*n* = 161) to evaluate construct validity of the Chinese ES. In exploratory factor analysis, we performed Kaiser-Meyer-Olkin (KMO) test and Bartlett’s test of sphericity to determine the feasibility of factor analysis. Then principal component analysis was used to explore the dimensionality of the ES. Confirmatory factor analysis was conducted to compute the model fit indices of the scale. The ratio of chi-square and degrees of freedom (CMIN/DF) between 1 and 3, root of the mean square residual (RMR) under 0.05, normed fit index (NFI), incremental fit index (IFI), Tucker-Lewis index (TFI), and comparative fit index (CFI) greater than 0.9 indicate that the model is goodness of fit [40]. The root mean square error of approximation (RMSEA) between 0.08 and 0.10 indicates that the model is acceptable and has a mediocre fit; the value between 0.05 and 0.08 means reasonable fit and if the value is less than 0.05, the model fit very well [41]. In order to further confirm the dimensionality and model fit indices of the scale, we used package in R (‘mirt’) to run Item Response Theory (IRT) model and M2 test and the results were shown in Additional file 2. The criterion validity, the extent to which the ES scores relate to a gold standard, was evaluated between the ES and PHQ-9 scores using Spearman’s correlation coefficient. The correlation of at least 0.70 with the gold standard is considered a positive rating for criterion validity [42]. Cronbach’s α and Spearman-Brown coefficients were used to evaluate the internal consistency reliability and split-half reliability, respectively. Usually 0.70 is recommended as a minimum standard for reliability [42].

We performed two hierarchical regression analysis to test unique associations of sociodemographic factors, entrapment with depression. In step 1, unique association of significant sociodemographic characteristics to depression was tested. The entrapment factor was added in the step 2, testing the increment in the explained variance of entrapment in the prediction of depression beyond sociodemographic factors [5, 7, 43]. The coefficient of determination, denoted R^2^, was used to indicate the proportion of the variance in the depression that is predictable from the sociodemographic and entrapment factors. Finally, receiver operator characteristic (ROC) curve analysis was performed to calculate optimal cut-off value of the ES for predicting depression [34]. The area under ROC curve (AUC) can measure the ability of an instrument to distinguish whether a subject has changed or not according to an external criterion [44]. An AUC of at least 0.70 is adequate [42]. *P* < 0.05 was considered statistically significant. All analyses were performed using SPSS 25.0, AMOS 24.0 and R software (version 3.6.1).

## Results

### Sociodemographic characteristics and sample comparisons

Table 1 presents an overview of participants’ sociodemographic characteristics and comparisons of the ES scores between subgroups. The age of participants ranged from 18 to 69 years, with a mean age of 29.91 years. Most (85.2%) participants were unmarried and 69.7% were self-identified homosexuals. The results suggested that only the difference of the ES scores between married and unmarried subgroups was statistically significant (*P* = 0.011).

**Table 1:** Sociodemographic characteristics and sample comparisons of Entrapment Scale scores (*n* = 304)

Sociodemographic Characteristics	Number of participants n (row%)	ES scores		***P*** value
		Mean ± SD	Median (IQR)	
**Age group**				0.791
< 25	82 (27%)	15.15 ± 14.14	11.5 (23)	
25–40	188 (61.8%)	14.19 ± 13.90	12 (22)	
41–59	31 (10.2%)	11.97 ± 11.73	8 (16)	
≥ 60	3 (1.0%)	11.00 ± 9.54	16 (17)	
**Education level**				0.512
Less than Junior high school	25 (8.2%)	16.08 ± 13.61	16 (27)	
High school	38 (12.5%)	13.13 ± 14.01	9.5 (17)	
Uni. /tech./prof.	241 (79.3%)	14.16 ± 13.70	11 (21)	
**Income**				0.232
≤ 3000	35 (11.5%)	17.23 ± 13.47	16 (26)	
3001–6000	84 (27.6%)	15.12 ± 14.35	12 (24)	
6001–12,000	114 (37.5%)	13.56 ± 13.57	10 (22)	
≥ 120,001	71 (23.4%)	12.59 ± 13.22	10 (17)	
**Marital status**				0.007
Married	35 (11.5%)	9.29 ± 14.07	3 (16)	
Unmarried	259 (85.2%)	14.73 ± 13.65	12 (22)	
Divorced and Widowed	10 (3.3%)	14.89 ± 8.59	16 (31)	
**Residence status**				0.521
Local	80 (26.3%)	13.99 ± 13.47	12 (21)	
Stayed ≤1 year	27 (8.9%)	16.63 ± 13.65	13 (24)	
Stayed 1–5 year(s)	109 (35.9%)	14.44 ± 13.83	10 (24)	
Stayed ≥5 years	88 (28.9%)	13.31 ± 13.91	10.5 (18)	
**HIV status**				0.956
Positive	9 (3.0%)	13.78 ± 13.60	14 (15)	
Negative	251 (82.5%)	14.18 ± 13.98	11 (21)	
Unknown	44 (14.5%)	14.34 ± 12.40	13 (24)	
**Self-reported sexual orientation**				0.052
Homosexuality	212 (69.7%)	15.47 ± 14.08	13.5 (22)	
Heterosexuality	9 (3.0%)	12.44 ± 16.46	3 (23)	
Bisexuality	69 (22.7%)	10.83 ± 11.63	6 (18)	
Not sure	14 (4.6%)	12.43 ± 13.95	8.5 (21)	

SD: standard deviation; IQR: inter-quartile range

### Construct validity

#### Exploratory factor analysis

The results showed that the KMO measure of sampling adequacy was 0.953 and the chi-square value of the Bartlett’s test was 2463.831 (*P* < 0.001), indicating the data were suitable for factor analysis. The principal component analysis with eigenvalues greater than 1 and covariance matrix demonstrated that a common factor was extracted and the percent of variance was 70.461%. The item communality and factor loading are shown in Table 2.

**Table 2:** Item communality and factor loading of the one-dimensional model (n = 143)

Item-Nr	Item	Communality	Factor Loading
1	I am in a situation I feel trapped in.	0.557	0.746
2	I have a strong desire to escape from things in my life.	0.716	0.846
3	I am in a relationship I can’t get out of.	0.623	0.789
4	I often feel like I would just like to run away.	0.764	0.874
5	I feel powerless to change things.	0.699	0.836
6	I feel trapped by my obligations.	0.715	0.845
7	I can’t see a way out of my current situation.	0.762	0.873
8	I would like to get away from other more powerful people in my life.	0.556	0.745
9	I strongly desire to leave and stay away from where I am now.	0.732	0.856
10	I feel trapped by other people.	0.652	0.807
11	I want to get away from myself.	0.673	0.820
12	I feel powerless to change myself.	0.673	0.820
13	I would like to escape from my thoughts and feelings.	0.763	0.873
14	I feel trapped inside myself.	0.790	0.889
15	I would like to get away from who I am and start again.	0.825	0.908
16	I feel I am in a deep hole that I can’t escape.	0.776	0.881

#### Confirmatory factor analysis

The results of exploratory factor analysis showed the ES should be regarded as a one-dimensional scale. However, the original scale was divided into two dimensions of internal entrapment and external entrapment. Therefore, the fit indices of one-dimensional and two-dimensional model were compared to determine the dimensionality of the ES. The results suggested the model fit indices of the one-dimensional model were all acceptable and better than two-dimensional model (Table 3). The regression coefficient of each item in one-dimensional model was statistically significant (Table 4).

**Table 3:** Model fit indices of the one-dimensional and two-dimensional model (n = 161)

Model fitting index	CMIN/DF	RMR	RMSEA	NFI	IFI	TLI	CFI
one-dimensional model	2.101	0.034	0.083	0.920	0.957	0.948	0.956
two-dimensional model	2.606	0.039	0.100	0.898	0.935	0.923	0.934

CMIN/DF: the ratio of chi-square and degrees of freedom; RMR: root of the mean square residual; RMSEA: root mean square error of approximation; NFI: normed fit index; IFI: incremental fit index; TLI: Tucker-Lewis index; CFI: comparative fit index

**Table 4:** Confirmatory factor analysis parameter estimation of the one-dimensional model (n = 161)

Item	Unnormalized parameter estimates	Standard error	t value	normalized parameter estimates
1	1.000	–	–	0.767
2	1.231	0.100	12.356*	0.870
3	1.032	0.116	8.876*	0.666
4	1.266	0.104	12.116*	0.857
5	1.357	0.108	12.520*	0.879
6	1.298	0.114	11.364*	0.815
7	1.259	0.108	11.642*	0.832
8	1.079	0.101	10.658*	0.776
9	1.243	0.106	11.761*	0.839
10	1.070	0.097	11.069*	0.799
11	0.990	0.092	10.706*	0.781
12	1.329	0.108	12.323*	0.870
13	1.120	0.094	11.977*	0.852
14	1.220	0.109	11.225*	0.809
15	1.208	0.106	11.431*	0.821
16	1.222	0.103	11.838*	0.843

*P < 0.001

#### Criterion validity

The ES total score was positively correlated with the PHQ-9 score (r = 0.756, *P* < 0.01). The Spearman’s correlation coefficient was good and statistically significant.

#### Reliability

The Cronbach’s α and Spearman-Brown coefficients were 0.970 and 0.976, respectively, suggesting the Chinese ES had good internal consistency reliability and split-half reliability.

#### Hierarchical regression analysis

Our data met the assumptions for multiple linear regression including measures for collinearity, and no multivariate outliers or influential cases. The hierarchical regression analysis was used to show the unique associations of sociodemographic factors, entrapment and depressive symptoms (Table 5). Significant sociodemographic characteristics including age, education level, marital status, income, residence status, HIV status and self-reported sexual orientation, tested in step 1, explained 4.8% (Adjusted R^2^ = 0.048, *P* < 0.01) of the variance in depression. Entrapment scores, added in step 2, significantly predicted an additional 45.1% (R^2^ Change = 0.451, *P* < 0.01) of depressive symptoms beyond effect of sociodemographic factors among the MSM population (β = 0.689, *P* < 0.001).

**Table 5:** Hierarchical regression analysis predicting depressive symptoms (Patient Health Questionnaire-9)

Model	R	R^2^	Adjusted R^2^	R^2^ Change	F value
Step1^a^	0.292	0.085	0.048	–	2.261*
Step2^b^	0.732	0.536	0.515	0.451	25.782*

^a^ independent variables included age, education level, marital status, income, residence status, HIV status, and self-reported sexual orientation

^b^ independent variables included age, education level, marital status, income, residence status, HIV status, self-reported sexual orientation, and ES score

R^2^: coefficient of determination

*P < 0.01

#### Sensitivity and specificity of the ES for predicting depression

**Fig. 1:**
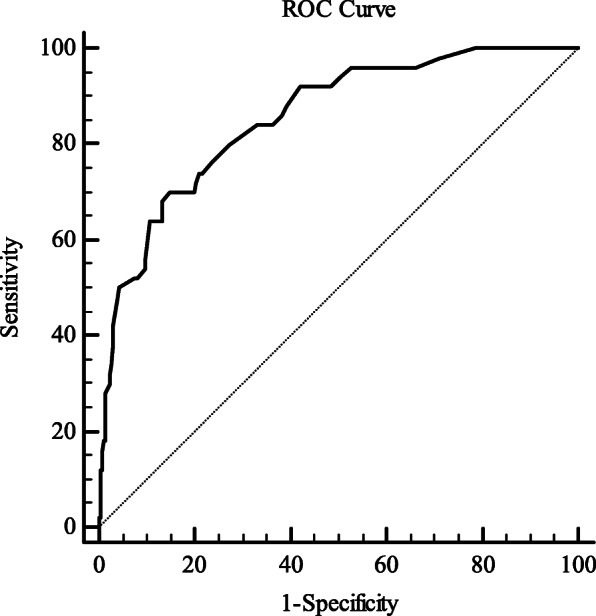
Receiver operator characteristic (ROC) curve for the Chinese entrapment scale as a predictor of depression. The area under the ROC curve was 0.854 (95% confidence interval: 0.809, 0.892). The optimum cut-off value of the ES for predicting depression was 23 with a sensitivity of 70% and specificity of 85.4% when a PHQ-9 score > 9 was considered to indicate depression

Fig. 1 shows the ROC for the ES as a predictor of depression. The ES had good value in predicting MSM with a PHQ-9 diagnosis of depression (area under the ROC curve was 0.854, 95% confidence interval: 0.809, 0.892). The optimum cut-off value of the ES for predicting depression was 23, which had sensitivity of 70% and specificity of 85.4% when a PHQ-9 score ≥ 10 was considered to be depressed.

## Discussion

The Chinese ES was shown to be a reliable instrument with high-level internal consistency and split-half reliability. The present results also confirmed that the ES was valid and feeling of entrapment was related to depression. Among the model fit indices, the value of RMSEA only reached an acceptable level. Previous researches pointed out model fit indices can be affected by sample size [45], degree of freedom (df) [46] and numbers of variables analyzed [47]. The denominator of the formula for RMSEA calculation contains both sample size and model df, which means the RMSEA value in complex model with high df estimated with large sample size can be decreased [48]. Accordingly, more participants can be recruited to calculate the model fit indices again especially RMSEA value in future studies.

The original ES consists of two subscales (EE and IE) because Gilbert and Allan thought the reasons for perceived entrapment are important. People may react in different ways to worse conditions, for example just perceiving of being trapped or wishing to escape from the situation. Theoretically, the strength of the escape motivation may be significant to the severity of depression [5]. A recent study also pointed out two separate dimensions were found to form the entrapment items and described as external and internal entrapment [3]. Robert et al. conducted confirmatory analyses supporting the two-factor solution of the ES is more reasonable [13]. However, factor analysis in this study demonstrated the ES can be considered as a single construct. This conclusion was consistent with previous studies that tested a German version of the ES and assessed the reliability and validity of the Chinese ES among medical students [7, 29]. Tucker et al. provided evidence that the model fit indices of a single factor solution of the ES are superior to that of two factor model in young adults [49]. These findings demonstrated that causes of entrapment were theoretically but not empirically divisible into internal and external sources. Some ES items cannot be easily distinguished as EE or IE, such as “I am in a situation I feel trapped in” or “I can’t see a way out of my current situation” [7]. Different studies draw different conclusions on the dimensionality of the ES. It is possible that the application of items is different from study to study, sample to sample, participant to participant. The most appropriate number of dimensions of the scale should be a balance between theory, model complexity and fit, clinical practice [3].

MSM are at high risk of depression. They are often not understood or accepted by the general public because of their sexual orientation and perceived sexual behavior and susceptibility to sexually transmitted diseases and mental health problems. Outside views and inner self-doubt and self-denial may have a mutual influence and association. MSM may therefore be unable to accurately divide self-perceived entrapment into internal or external sources. Our data showed unmarried MSM had higher ES scores than the married group. In China, homosexual marriage has not been recognized. Heterosexual marriage is possible to help MSM conceal their sexual orientation and avoid social criticism and pressure. Depression is considered to be a complicated combination of high negative affectivity and low positive affectivity [50]. The correlation coefficient between the ES and PHQ-9 scores was 0.756 (*P* < 0.01), and the former explained nearly half of the variance in the later. This reflected entrapment as a relevant and distinct construct in explaining depression in the MSM population. A cutoff score of 23 on the ES was suggested to be optimal (with a sensitivity of 70% and a specificity of 85.4%) when predicting a diagnosis of depression as measured by the PHQ-9. Therefore, the MSM population can rate their own perceptions and judgments about entrapment and pay attention to their current mental state using this ES cutoff score. However, this cut-off score should be used with caution in screening and other populations. The conceptualization of a state of entrapment implies that feeling of entrapment may change over time [7, 51]. If an individual is measured with high score for multiple times in the short- or long-term, their mental health problems may merit attention.

Entrapment has also been associated with anxiety, anhedonia, feeling of shame, hopeless, and suicide ideation [14, 52, 53]. It is important that MSM recognize the perception of entrapment early to allow timely implementation of psychological and suicide prevention interventions to avoid or relieve depressive symptoms and suicide ideation. Improving self-cognition and self-affirmation and enhancing self-defensive ability and external support resources are of value for MSM [54]. In addition, increased social acceptance and support may enhance self-perceived social status and improve coping ability when facing outside threats, stress, and criticism [55]. Screening for psychological status and comprehensive interventions integrating psychology, society, and behavior need to be strengthened in primary care settings.

### Limitations

There were some limitations in this study. First, participants might have had some concerns when completing the questionnaire because of privacy issues, which could have resulted in information bias. However, all investigators have participated in trainings and an anonymous, self-administered questionnaire was used to maximize the data quality. Second, the MSM population in the study setting is small and relatively hard to reach, meaning strict random sampling was impossible for this study. The snowball sampling method used in this study inevitably produces selection bias and sample representation problems. However, snowball sampling can identify more subjects that meet study requirements relatively easily at low cost and high efficiency. Third, only one scale of depression was evaluated in this study. Maybe other scales of depression can be added in the further study to get a more accurate understanding of depression in the MSM population. Another limitation is that ES was not given a second time in the same individuals due to the specificity and anonymity of the MSM population, hence the test-retest reliability cannot be measured. Finally, participants were limited to the MSM population in Shanghai, which is an economically developed and culturally open city; the social acceptance of MSM may be higher than in other areas. Therefore, this sample cannot represent all MSM populations in China. Use of the ES should be further explored in other areas of China.

## Conclusions

The MSM population is at high risk for depression and suicide ideation, which suggests that close attention should be paid to mental health problems among this group, especially psychological problems related to depression (e.g., feeling of entrapment). The Chinese version of the ES has good psychometric properties and can be extended to all MSM populations in China to evaluate perception of entrapment. This will support early identification and early intervention for mental health problems in this population.

## Supplementary Information

**Additional file 1.**

**Additional file 2.** The historgram of the entrapment scale scores and the IRT model and M2 test of the entrapment scale among men who have sex with men in Shanghai, China.

## Data Availability

The datasets used and analyzed during the current study are available from the corresponding author on reasonable request.

## Abbreviations

MSM: men who have sex with men
ES: entrapment scale
EE: external entrapment
IE: internal entrapment
PHQ-9: Patient Health Questionnaire-9
SD: standard deviation
IQR: inter-quartile range
CMIN/DF: the ratio of chi-square and degrees of freedom
RMR: root of the mean square residual
RMSEA: root mean square error of approximation
NFI: normed fit index
IFI: incremental fit index
TLI: Tucker-Lewis index
CFI: comparative fit index
ROC: receiver operator characteristic
AUC: under ROC curve
