# Supplementary material for: Evaluating the validity and reliability of the Chinese entrapment scale and the relationship to depression among men who have sex with men in Shanghai, China
Source: BMC Psychiatry. 2021 Jul 2;21:328. doi: 10.1186/s12888-021-03333-9 (PMC8254295; doi:10.1186/s12888-021-03333-9)
Supplement: Supplementary file 1 — Additional file 1. [file 12888_2021_3333_MOESM1_ESM.docx]

**困顿感量表**

**Entrapment Scale**

**评分:** 0 = 没有 1 = 很轻 2 = 中等 3 = 偏重 4= 严重

**Score:** 0 = Not at all 1 = A little bit 2 = Moderately 3 = Quite a bit 4 = Extremely

| **条目**  **Item** | **中文版**  **Chinese version** | **英文版**  **English version** |
| --- | --- | --- |
| 1 | 我处在困顿的状态。 | I am in a situation I feel trapped in. |
| 2 | 我强烈希望逃避我的生活。 | I have a strong desire to escape from things in my life. |
| 3 | 我处在一段无法摆脱的关系中 | I am in a relationship I can’t get out of. |
| 4 | 我经常觉得我就想逃离。 | I often feel like I would just like to run away. |
| 5 | 我对改变事物很无力。 | I feel powerless to change things. |
| 6 | 我感到被自己的义务所困。 | I feel trapped by my obligations. |
| 7 | 我看不到摆脱目前状态的出路。 | I can’t see a way out of my current situation. |
| 8 | 我想要离开生活中其他比我强的人。 | I would like to get away from other more powerful  people in my life. |
| 9 | 我强烈希望离开并远离我现在生活的  地方。 | I strongly desire to leave and stay away from  Where I am now. |
| 10 | 我认为我被别人困住了。 | I feel trapped by other people. |
| 11 | 我想要远离我自己。 | I want to get away from myself. |
| 12 | 我对改变自己很无力。 | I feel powerless to change myself. |
| 13 | 我想要逃避我的想法和感受。 | I would like to escape from my thoughts and  feelings. |
| 14 | 我觉得我被自己困住了。 | I feel trapped inside myself. |
| 15 | 我想要远离自我重新开始。 | I would like to get away from who I am and start  again. |
| 16 | 我觉得我在一个无法逃离的深洞里。 | I feel I am in a deep hole that I can’t escape. |
