# Supplementary material for: Evaluating the validity and reliability of the Chinese entrapment scale and the relationship to depression among men who have sex with men in Shanghai, China
Source: BMC Psychiatry. 2021 Jul 2;21:328. doi: 10.1186/s12888-021-03333-9 (PMC8254295; doi:10.1186/s12888-021-03333-9)
Supplement: Supplementary file 2 — Additional file 2. The historgram of the entrapment scale scores and the IRT model and M2 test of the entrapment scale among men who have sex with men in Shanghai, China. [file 12888_2021_3333_MOESM2_ESM.docx]

Figure 1 the Frequency of the Entrapment Scale Scores among Men Who Have Sex with Men in Shanghai, China

IRT model and M2 test

The IRT model was constructed for the 16 items of the ES. Full-information item factor analysis showed that only one factor was extracted, corresponding with the results through exploratory and confirmatory factor analyses. The coefficients, F1 and h2 values were summarized in the table 1 and table 2 below. The SS loading and proportion variance were 8.754 and 0.584, respectively. Subsequently, the M2 test was evaluated and the results were showed in the table 3. The conclusions are basically corresponding with the results in the article.

Table 1 The coefficients of IRT model of the Entrapment Scale among Men Who Have Sex with Men in Shanghai, China

| Item | a1 | d1 | d2 | d3 | d4 |
| --- | --- | --- | --- | --- | --- |
| 1 | 2.767 | 1.218 | -1.691 | -5.132 | -7.076 |
| 2 | 4.287 | 0.671 | -3.223 | -5.681 | -10.726 |
| 3 | 2.172 | -0.238 | -1.969 | -3.509 | -5.123 |
| 4 | 4.037 | 0.961 | -2.657 | -5.234 | -8.633 |
| 5 | 4.333 | 1.493 | -2.638 | -5.507 | -8.04 |
| 6 | 3.146 | 0.688 | -1.994 | -4.091 | -6.177 |
| 7 | 3.307 | 0.857 | -1.769 | -4.646 | -7.043 |
| 8 | 3.3 | -0.515 | -3.74 | -5.397 | -6.603 |
| 9 | 3.842 | 0.126 | -3.252 | -5.436 | -7.489 |
| 10 | 3.317 | -0.421 | -3.641 | -5.302 | -7.407 |
| 11 | 3.266 | -1.228 | -3.811 | -6.494 | -7.131 |
| 12 | 4.278 | 0.381 | -3.111 | -5.848 | -7.878 |
| 13 | 4.686 | -0.578 | -3.825 | -8.069 | -10.391 |
| 14 | 3.457 | -0.272 | -2.592 | -5.007 | -7.21 |
| 15 | 3.432 | 0.094 | -2.545 | -5.061 | -7.592 |
| 16 | 4.524 | -0.975 | -3.792 | -7.074 | -8.467 |

Table 2 The summary of IRT model of the Entrapment Scale among Men Who Have Sex with Men in Shanghai, China

| Item | F1 | h2 |
| --- | --- | --- |
| 1 | 0.852 | 0.725 |
| 2 | 0.929 | 0.864 |
| 3 | 0.787 | 0.619 |
| 4 | 0.921 | 0.849 |
| 5 | 0.931 | 0.866 |
| 6 | 0.880 | 0.774 |
| 7 | 0.889 | 0.791 |
| 8 | 0.889 | 0.790 |
| 9 | 0.914 | 0.836 |
| 10 | 0.890 | 0.792 |
| 11 | 0.887 | 0.786 |
| 12 | 0.929 | 0.863 |
| 13 | 0.940 | 0.883 |
| 14 | 0.897 | 0.805 |
| 15 | 0.896 | 0.803 |
| 16 | 0.936 | 0.876 |

Table 3 M2 test of the Entrapment Scale among Men Who Have Sex with Men in Shanghai, China

| M2 | df | p | REMSA | RMSEA_5 | RMSEA_95 | SRMSR | TLI | CFI |
| --- | --- | --- | --- | --- | --- | --- | --- | --- |
| 80.267 | 56 | 0.018 | 0.052 | 0.022 | 0.076 | 0.056 | 0.986 | 0.989 |
